# The Effect of Severe Intraventricular Hemorrhage on the Biorhythms of Feeding in Premature Infants

**DOI:** 10.3389/fped.2021.673152

**Published:** 2021-08-23

**Authors:** Ira H. Gewolb, Babatunde T. Sobowale, Frank L. Vice, Abhijit Patwardhan, Nino Solomonia, Eric W. Reynolds

**Affiliations:** ^1^Division of Neonatology, Department of Pediatrics and Human Development, Michigan State University, East Lansing, MI, United States; ^2^Department of Biomedical Engineering, University of Kentucky, Lexington, KY, United States; ^3^Division of Neonatology, Department of Pediatrics, University of Kentucky School of Medicine, Lexington, KY, United States; ^4^M. Iashvili Childrens Central Hospital, Tbilisi, Georgia; ^5^McGovern Medical School, University of Texas Health Science Center at Houston, Houston, TX, United States

**Keywords:** infant feeding, biorhythms, suck, swallow, premature infant, intraventricular hemorrhage, nutritive suck, deglutition disorders

## Abstract

**Background:** Suck-swallow rhythmicity and the integration of breathing into infant feeding are developmentally regulated. Neurological injury and breathing abnormalities can both impact feeding in preterm infants.

**Objective:** To determine the effects of neurologic injury independent of effects of disordered breathing on feeding biorhythms in premature infants.

**Methods:** Low-risk preterm infants (LRP), infants with Grade 3–4 Intraventricular Hemorrhage (IVH), those with bronchopulmonary dysplasia (BPD), and those with both BPD and IVH (BPD+IVH) were identified. Forty-seven infants, 32–42 weeks Postmenstrual Age (PMA) were evaluated on one or more occasions (131 studies). Of these, 39 infants (81 studies) were performed at >35 weeks PMA. Coefficient of variation (COV) (=standard deviation of the inter-event (e.g., suck-suck, swallow-breath, etc.) interval divided by the mean of the interval) was used to quantify rhythmic stability.

**Results:** To adjust for PMA, only those infants >35–42 weeks were compared. Suck-suck COV was significantly lower (more rhythmically stable) in the LRP group [COV = 0.274 ± 0.051 (S.D.)] compared to all other groups (BPD = 0.325 ± 0.066; IVH = 0.342 ± 0.072; BPD + IVH = 0.314 ± 0.069; all *p* < 0.05). Similarly, suck-swallow COV was significantly lower in LRP babies (0.360 ± 0.066) compared to the BPD group (0.475 ± 0.113) and the IVH cohort (0.428 ± 0.075) (*p* < 0.05). The BPD+IVH group (0.424 ± 0.109), while higher, was not quite statistically significant.

**Conclusions:** Severe IVH negatively impacts suck-suck and suck-swallow rhythms. The independent effect of neurological injury in the form of IVH on feeding rhythms suggests that quantitative analysis of feeding may reflect and predict neurological sequelae.

## Background

Biorhythmic stability and the coordination/integration of rhythmic suck-swallow-respiration characterize efficient oral feeding in infants. Establishment of the underlying rhythms of suck and swallow follows a predictable and quantifiable developmental pattern in preterm infants and has been shown to correlate with postmenstrual age (PMA) rather than postnatal age (PNA) (1–3). This suggests that oral feeding is an innately programmed rather than a learned behavior (1–3). Understanding deviations from the normal underlying biorhythms of infant suckle feeding may yield insights into the neuro-developmental maturation of the neonate, especially the development of the brainstem, where respiratory and swallow centers are located (4).

The most complex human neuromuscular unit in the body is the upper aerodigestive tract, which acts as conduit for passage of solids, liquids, and gas (5). Normal sequencing of sucking, swallowing, and breathing requires integration of multiple afferent and efferent systems in the central nervous system. This complex interaction can be altered or modified by insults to the central nervous system (6–13).

We have previously shown that the suck-swallow-breath rhythms and patterns in preterm infants with bronchopulmonary dysplasia (BPD) differ significantly from those in low-risk preterm infants (14–18). This has raised the question as to whether the dysrhythmias and abnormal coordination patterns noted in infants with BPD are caused by difficulty breathing or by the often concomitant underlying neurological injury commonly seen as co-morbid conditions (such as IVH) in medically complex preterm infants. Neonates with BPD often have IVH or other markers of neurological injury; thus, it is important to differentiate the dysrhythmic effects of neurological injury from any direct respiratory effects of BPD on infant feeding, since a specific “signature” in feeding in infants with neurological issues pointing to a more vulnerable population would allow resources to be targeted for such children.

## Objective

Our objective was to evaluate the underlying rhythms of suck, swallow, and breath in a low-risk cohort of preterm infants, as well as in cohorts with severe IVH, BPD, or BPD + IVH, thus allowing us to determine whether neurological injury alone has an adverse impact on the rhythms of infant feeding.

We hypothesized that the attainment of rhythmic stability of suck-suck and suck-swallow dyads would be adversely impacted in the high-risk preterm groups and that respiratory and neurological issues might have different effects on the overall biorhythmic patterns seen.

## Methods

### Population

A convenience sample of 47 preterm infants [gestational age (GA) at birth = 23 5/7–35 weeks] (131 studies) were studied at the University of Kentucky Neonatal Intensive Care Unit (NICU) from May 2007-October 2011. Infants were evaluated on one or more occasions between 32 and 42 weeks PMA using bottle feeds. All infants <36 weeks at birth were eligible; exclusion criteria were concurrent sepsis, craniofacial anomalies, or a history of maternal drug-abuse. No such infants were included in the study.

The first analysis used all 47 infants (131 studies) to ascertain that low-risk preterm (LRP) infants (*n* = 21; 53 studies) did indeed follow a developmentally regulated pattern of maturation of suck-swallow rhythmic stability (lower interval COV) over time, as opposed to the other 3 groups (BPD, *n* = 10; 22 studies), IVH (*n* = 7; 24 studies) and IVH + BPD (*n* = 9; 32 studies).

To correct for the potential confounding effect of PMA, since most of the studies performed before 35 weeks post-menstrual age (PMA) were in the LRP with only 3 in the BPD group, 5 in the IVH group and 8 in the IVH + BPD group, we then studied only those 39 infants with studies performed after 35 weeks PMA. There were 13 such LRP control infants (19 studies), 10 infants with BPD (19 studies), 7 infants with IVH Grade 3–4 but without BPD (19 studies), and nine babies with IVH and BPD (24 studies).

LRP infants were born prior to 35 completed weeks of gestation, did not have IVH Grade 3 or 4, had no congenital anomalies, and did not develop BPD. BPD was defined as a need for oxygen at ≥ 36 weeks PMA. All premature infants were routinely screened for IVH, generally at day 3 and 7 of life. Severe IVH was defined as Grade 3–4 (19).

### Methods

Infants were generally studied weekly beginning shortly after (1–5 days) the time of initiation of oral feeds until discharge, with the timing of initial feeding determined by the medical team caring for the infant (usually 32–35 weeks PMA).

As previously described, an open-ended 5 French nasopharyngeal catheter was placed and connected to a pressure transducer (Transpac IV Neonatal/Pediatric Pressure Monitoring Kit, Hospira Inc., Lake Forrest, IL) to measure pharyngeal swallow pressure. The nasopharyngeal catheter was placed at the back of the pharynx by measuring the distance from the nose to the lower portion of the ear and then to the angle of the mandible. It was learned through early trials (1, 20) that this placement was the optimal position for recording changes in pressure with swallow.

A second catheter was placed through the nipple of the bottle so that the catheter tip was flush with the nipple and connected to a transducer to measure suck pressure. This method only measures pressure changes (suction) and does not identify the expression component of suckle.

Thoracic respiratory effort was measured plethysmographically with a stretchable strain gauge placed around the infant's chest (Pneumotrace II, Model 1132, UFI, Morro Bay, CA). Being 1.5 inches wide, the band usually covers the area from the baby's nipple line to the mid-epigastric area.

Nasal airflow was measured with a small thermistor bead (Omega 44030, Omega Engineering Inc., Stamford, CT) in a custom assembly placed at the opening of the naris.

Leads and transducers were placed approximately one-half hour prior to a feed. Infants were bottle fed their regular formula or breastmilk during their normally scheduled feeding time.

Data were collected and displayed as linear graphs using the Windaq Acquisition System and Waveform Browser (Dataq Industries, Akron, OH).

The coefficient of variation (COV) [the standard deviation of the inter-event (suck-suck, swallow-swallow, suck-swallow, swallow-breath) interval divided by the mean of the interval] was used to quantify rhythmic stability. Only “runs” of ≥ 3 swallows were analyzed. Apneic swallows were defined as ≥ 3 swallows without interspersed respirations.

Statistical analyses [ANOVA (with ranks, where appropriate)] with Dunnett's *post-hoc* test of high-risk categories vs. controls and regression analyses were performed using SigmaStat 3.5 (Systat Software, Inc, 2107 San Jose, CA). *P* < 0.05 was considered statistically significant. This study was approved by the Institutional Review Boards of the University of Kentucky School of Medicine and Michigan State University College of Human Medicine. Written parental informed consent was obtained prior to studying each infant.

## Results

**Figure 1:**
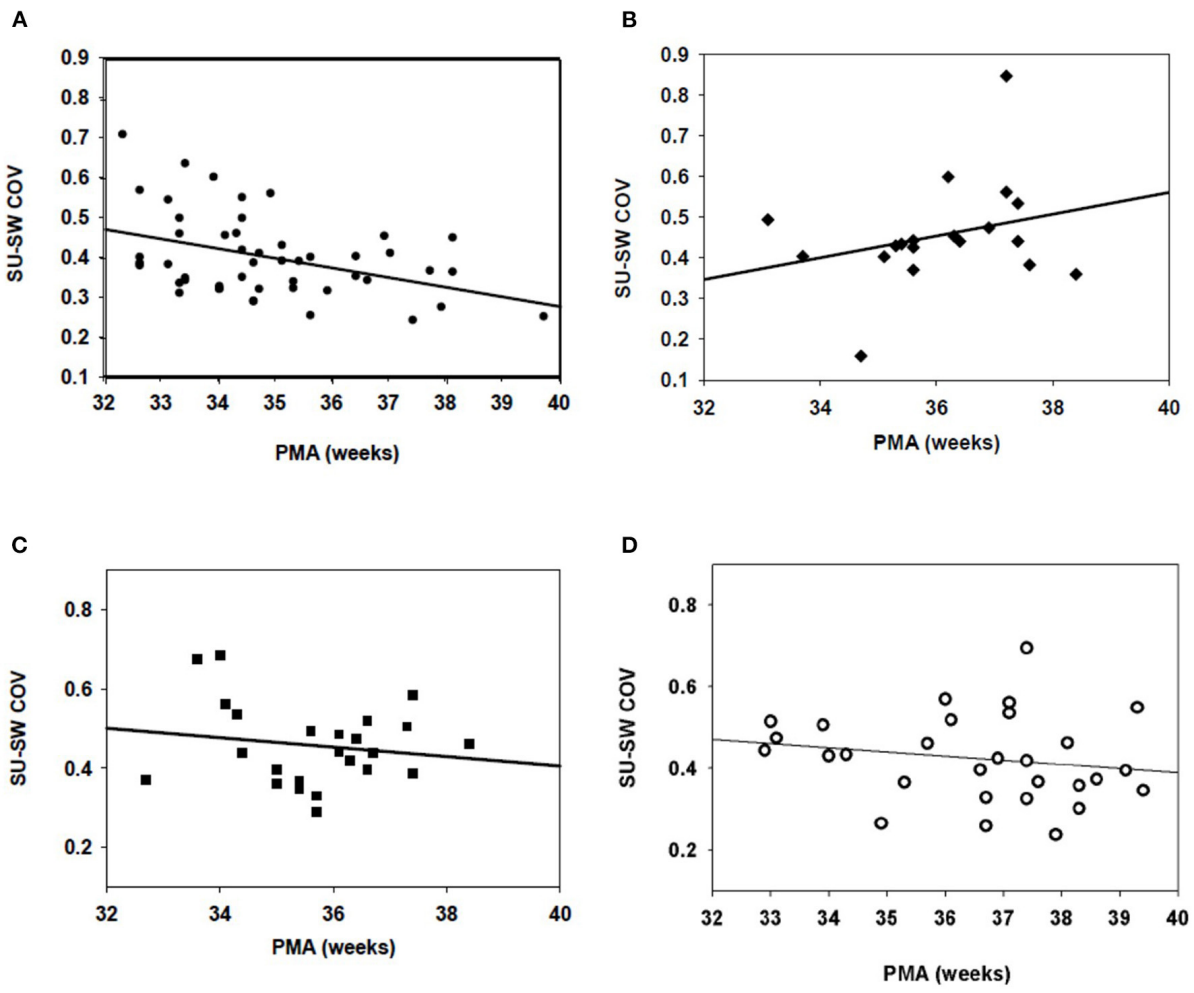
Suck-swallow (SU-SW) dyadic COV in infants 32–40 weeks PMA. **(A)** In 21 low risk preterm (LRP) infants (53 individual studies), there was a statistically significant (*p* > 0.05) inverse regression between Suck-Swallow COV and 32–40 weeks PMA (*R* = 0.407). **(B)** In 10 infants with BPD (22 feeds) there was no correlation between time from 32 to 40 weeks and suck-swallow COV. *R* = 0.181; *p* = NS). **(C)** In babies with IVH (but no BPD), *N* = 7 (24 studies), there was no improvement in Suck-Swallow COV between 32 and 40 weeks PMA (*R* = 0.161; *p* = NS). **(D)** In infants with both IVH and BPD (*N* = 9; 32 studies), there was also no further improvement in Suck-Swallow COV between 32 and 40 weeks PMA (*R* = 0.184; *p* = NS).

When all studies from 32 to 42 weeks were analyzed, the LRP control group demonstrated a clear correlation (*R* = 0.407) between decreasing suck-swallow COV and post-menstrual age (Figure 1A); *p* < 0.004. There was no significant change over time in the BPD group (*R* = 0.181; *p* = NS) (Figure 1B). Of note, no significant improvement over time was found in either the IVH (*R* = 0.161; *P* = NS) (Figure 1C) or the IVH + BPD (*R* = 0.184; *p* = NS) groups Figure 1D). All groups began at a suck-swallow COV of ~0.400–0.450 and ended at 0.400–0.450 except for the LRP group that decreased over time to slightly < 0.300 COV (Figure 1A).

As noted above, there were very few studies in the high-risk groups that were performed at <35 completed weeks PMA. To avoid the inherent bias introduced by the PMA confounder, for the subsequent part of the study we only utilized infants who had studies performed after 35 weeks PMA.

As can be seen in the Table 1 population demographics for this group, gestational age at birth was significantly lower in infants with BPD and BPD + IVH compared to controls (and IVH). Birthweight was also lower in infants with BPD compared to the control and IVH groups. Of importance, PMA at the time of study was similar in the control, BPD and IVH groups, and was *higher* in infants with BPD + IVH; if anything, this would make it *less* likely to see a difference in rhythmicity between LRP and the *older* IVH+BPD cohort. Also, as expected, post-natal age at the time of the study was greater in the BPD and BPD + IVH groups compared to controls (Table 1).

**Table 1:** Population characteristics.

		**LRP**	**BPD**	**IVH**	**BPD + IVH**	
*N* =	Infants/studies	13/19	10/19	7/19	9/24	–
GA weeks	total	28.6 ± 2.6	26.0 ± 1.3^*^	29.2 ± 1.8	25.8 ± 1.2^*^	^*^P <0.05; LRP vs. BPD, BPD + IVH
BW grams	total	985 ± 217	751 ± 164^*^	1,155 ± 232	821 ± 323	^*^P <0.05; LRP vs. BPD
PMA	≥35 weeks	36.6 ± 1.3	37.0 ± 1.2	36.3 ± 0.9	37.7 ± 1.4^*^	^*^P <0.05; LRP vs. BPD + IVH
PNA	≥35 weeks	54.3 ± 19.8	79.6 ± 12.0^*^	47.9 ± 13.3	83.0 ± 13.1^*^	^*^P < 0.05; LRP vs. BPD, BPD + IVH

*PMA and PNA are only given for those babies with studies done at >35 weeks PMA*.

*Data given as mean ± S.D.*

* *P < 0.05 vs. LRP*.

**Figure 2:**
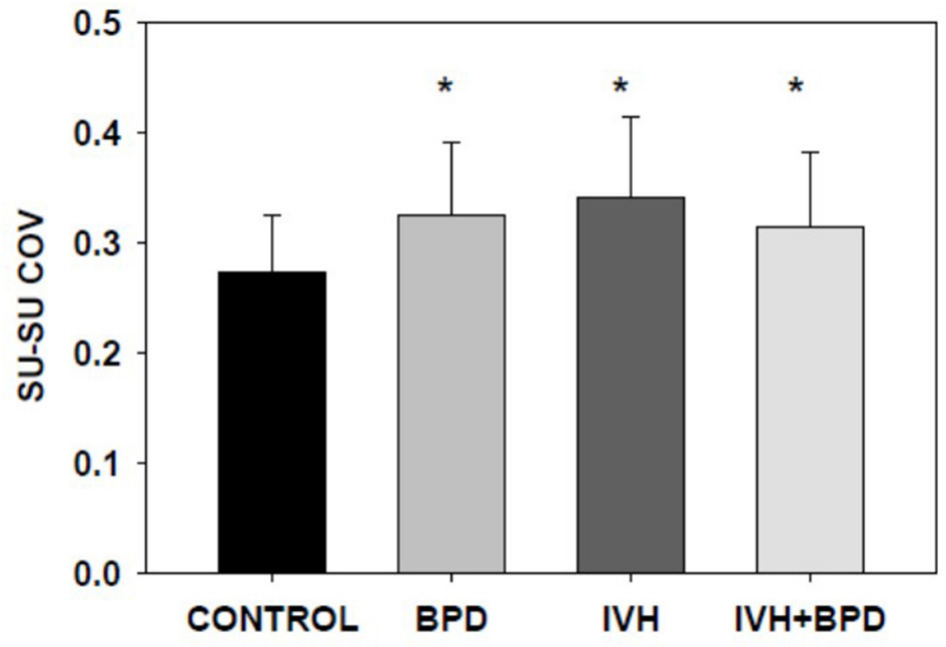
Suck-swallow (SU-SW) dyadic COV at >35 weeks PMA. In premature infants 35–42 weeks PMA, the stability of suck-swallow rhythm is greater (lower coefficient of variation) in control LRP infants compared to infants with BPD, IVH, and IVH + BPD (*p* < 0.05; ANOVA; Dunnett's *post-hoc* test). *p < 0.05 vs. LRP. Data is given as mean ± S.D.

**Figure 3:**
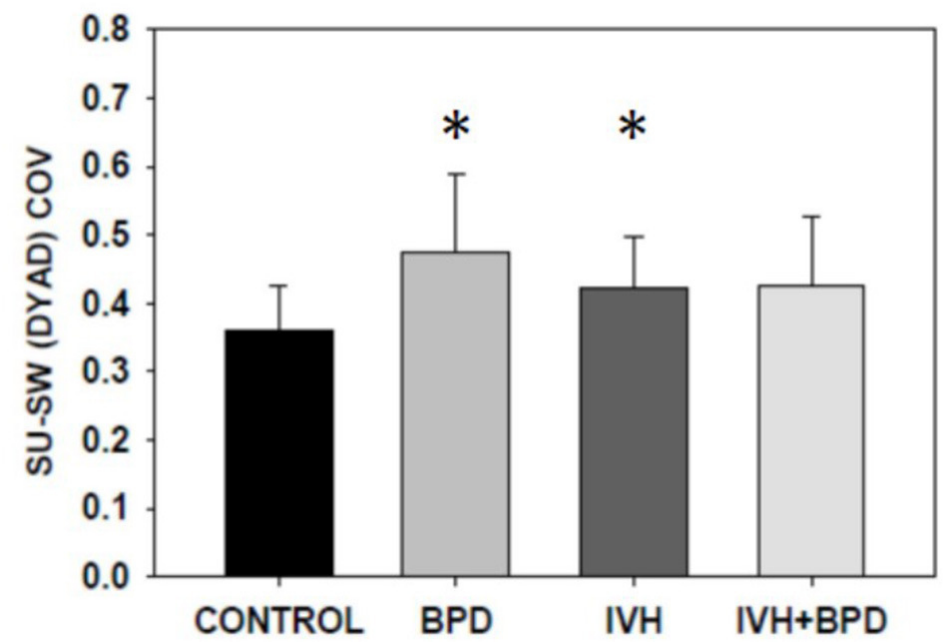
Suck-suck COV at >35 weeks PMA. In premature infants 35–42 weeks PMA, suck-suck rhythm is more stable in control LRP infants compared to infants with BPD and those with IVH (*p* < 0.05; ANOVA, Dunnet's *post-hoc* test). Infants with both IVH and BPD approached significance (*p* = 0.1). No additive or synergistic effect was noted. *p < 0.05 vs. LRP. Data is given as mean ± S.D.

In the 35–42 week PMA cohort, the suck-suck COV was significantly lower (had greater rhythmic stability) in the LRP group compared to all other groups (*p* < 0.05) (Figure 2). Also, the dyadic (suck-swallow) COV was significantly more stable (*lower* COV) in the LRP group (*p* < 0.05 vs. the IVH and the BPD groups) and approached significance (*p* = 0.1) vs. the BPD + IVH cohort (Figure 3).

LRP infants had shorter swallow intervals (higher swallow rates) than did infants with BPD (*p* < 0.05) (Table 2). Swallow-swallow COV was *lower* (greater rhythmic stability) in the BPD group than in the IVH group (Table 2). There were no significant differences in % apneic swallows (although, the average apneic rates were higher in the 3 high-risk groups) or in swallow-breath COV.

**Table 2:** Feeding characteristics.

	**LRP**	**BPD**	**IVH**	**BPD + IVH**	
% SU-run	71.1 ± 14.6	61.5 ± 20.0	64.5 ± 12.9	77.5 ± 11.1	NS
SU-interval	1.058 ± 0.147	0.981 ± 0.218	1.023 ± 0.129	1.038 ± 0.129	NS
SU-SU COV	0.274 ± 0.051	0.325 ± 0.066^*^	0.342 ± 0.072^*^	0.314 ± 0.069^*^	^*^P <0.05; LRP vs. BPD, IVH, BPD + IVH
SU per run	7.24 ± 6.51	5.23 ± 2.91	5.05 ± 3.06	7.84 ± 4.54	NS
% SW-run	0.627 ± 0.197	0.485 ± 0.285	0.518 ± 0.177	0.637 ± 0.206	NS
SW-interval	1.212 ± 0.092	1.358 ± 0.154^*^	1.243 ± 0.090	1.202 ± 0.124	^*^P <0.05; LRP vs. BPD
SW-SW COV	0.240 ± 0.037	0.202 ± 0.037^∞^	0.257 ± 0.036	0.231 ± 0.053	^∞^*P* <0.05; IVH vs. BPD
SW per run	7.80 ± 7.49	4.24 ± 2.85^*^	4.32 ± 1.74	7.15 ± 5.61	NS
SU-SW (dyad) COV	0.360 ± 0.066	0.475 ± 0.113^*^	0.423 ± 0.075^*^	0.424 ± 0.104	^*^P <0.05; LRP vs. BPD, IVH
SW-BR COV	0.354 ± 0.129	0.368 ± 0.150	0.305 ± 0.073	0.322 ± 0.107	NS
% apnea > 3	17.2 ± 15.5	27.6 ± 21.2	24.2 ± 23.5	29.5 ± 24.7	NS

*Suck-swallow-breath analysis: significant differences were found for all three pathologic groups vs. LRP for SU-SU COV; for BPD vs. LRP for SW-SW COV; and for BPD and IVH vs. LRP for SU-SW COV*.

*In addition, BPD infants had a higher swallowing rate (shorter SW-SW interval) vs. the IVH group. Data given as mean ± S.D.*

* *p < 0.05 vs. LRP*.

∞ *p < 0.05 BPD vs. IVH*.

## Discussion

Improvement in suck/swallow biorhythms correlate with increasing postmenstrual age (but *not* with increasing post-natal age) in a quantitative and predictable manner (1, 3, 20). Our previous results indicated that suck-suck COV, % suck -runs, sucks per run, and decreasing suck-intervals correlated with increasing PMA, as did % swallow -runs. Suck-swallow dyadic COV and % apneic runs also improved with increasing PMA (1). Indeed, the finding of decreasing suck-swallow dyadic COV is a clear validation of our previous findings (14).

Our previous results (14–18) indicated that infants with BPD had increased suck-suck COV (decreased rhythmic stability), decreased aggregation into suck runs, and shorter suck runs compared to controls. There was a decrease in swallow-swallow COV, % swallow runs and swallows per run in the BPD group. Suck-swallow dyadic COV was increased in the BPD group. There was no significant change over time in suck-swallow COV in the BPD group, in keeping with our current results (Figure 1B). Swallow-breath COV was also increased. The differences in these parameters followed the same pattern in the current study, although significance was not always attained.

Prior studies did not separate out infants with neurological injury, so the current study adds the finding that severe IVH alone, even in those babies without BPD, is sufficient to be associated with changes in the biorhythms of infant feeding. Thus, the differences in rhythmic stability (suck-suck COV and suck-swallow dyadic COV) in the children with IVH compared to controls appear to indicate that the abnormalities seen in IVH are not the result of dyspnea caused by BPD, and thus lend support to a relation between the biorhythms of feeding and neurological injury.

GA at birth was greater in the LRP group compared to the BPD and BPD+IVH cohorts. However, the PMA was greater in the BPD + IVH group vs. the LRP infants. This would imply that the high-risk infants were, on average, actually *more* mature at the time of their studies; this would tend to *lessen* any differences between these groups and the LRP group. This makes the fact that some critical measures were better in the low-risk group even more remarkable, since PMA (but not PNA) at time of study has been shown to correlate with rhythmic measures of maturity (1, 3).

The centers for suck, swallow and respiration control are located in the brainstem (4). We recognize that ultrasound techniques used to visualize IVH are not the optimal modalities to identify brainstem injury. Indeed, general white matter injury might be a better representation of neurological injury in preterms. However, this would have required more invasive imaging (MRI) of each of the infants in the study. This was not part of the original study design, which was meant to be as non-invasive as possible. Each child was studied ultrasonographically twice in the first week of life and once at ~28 days to assess for periventricular leukomalacia. Further MRI imaging was only done by the clinical team for suspicion of post-hemorrhagic hydrocephalus or seizures, but the “*n*” was not large enough to do an analysis or to subdivide the IVH group meaningfully. Therefore, since it is well-known that hypoxic injury contributes to the pathogenesis of IVH (13), we chose to use IVH as a surrogate for more global hypoxic injury.

Poor feeding behaviors have been associated with abnormal neurodevelopmental outcome (21), but only a few papers have focused specifically on sucking behavior (6–12, 22). In general, these studies have used qualitative feeding outcome measures (like the Neonatal Oral-Motor Assessment Scale- NOMAS), and not quantitative motions like the biorhythms of suck-suck or suck-swallow. While the NOMAS tool has a number of different assessments of sucking efficacy, most do not seem to correlate with long-term outcome. For example, Wolthius-Stigler et al. (11) found that only inability to start sucking at 44 weeks or inability to sustain sucking at 46 weeks PMA (i.e., babies with already established feeding difficulties, but not necessarily neurologically associated problems) yielded positive correlations with longer term feeding outcomes. Indeed, this points out another potential difference with the use of NOMAS–some of its measures may depend as much on muscle strength as on neurological injury. Medoff-Cooper et al. (23) relied on a single 5-min exam at term PMA on which to base their comparisons. By contrast, our use of rhythmicity can be quantitated, is less subjective and can be performed earlier than NOMAS, perhaps identifying infants at risk sooner in order to allow for earlier interventions. Furthermore, our studies are all performed prior to 44 weeks PMA, and so are not comparable to NOMAS. Thus, our data only reveal an early association/correlation with acute IVH and not with long-term neurological outcomes; further research is required to delineate the value of biorhythmic analysis in predicting later motor outcomes and to establish reliable measures of early sucking and swallowing function.

The link between early feeding difficulty and long-term neurodevelopmental outcomes is somewhat contentious. Of the published results most authors have found early feeding problems are associated with neurologic injury and poor long-term outcomes, but a few have found no link between them. Outcome measures also vary among the published studies including specific diagnoses (for example, cerebral palsy), scores on the Bayley Scales of Infant Development and speech, and language or cognitive assessments. Slattery et al. (21), published a review of the literature regarding early sucking and swallowing problems as predictors of neurodevelopmental outcomes. In their review, they found nine studies, prior to 2012, examining the relation between early sucking and swallowing problems and various aspects of neonatal brain injury, including a mixture of prospective and retrospective cohort studies and case series. They concluded that “there is currently insufficient evidence to clearly determine the relation between early sucking and swallowing problems and neonatal brain injury.”

The current study does not address long-term outcome, but rather focuses on the association between early feeding assessment and acute neurological injury. Nevertheless, the correlation of biorhythms with acute neurological injury may open an earlier window with which to track and perhaps direct resources to babies at higher risk for adverse long-term outcomes.

## Conclusion

Severe IVH has a negative impact on the biorhythms of suck-suck and suck-swallow in preterm infants 35–42 weeks PMA. If a preterm infant with IVH but without BPD at 35–42 weeks PMA lacks adequate feeding biorhythms, there could be a need for additional workup to identify possibly undetected neurological injury. The independent effect of severe IVH on feeding rhythms suggests that quantitative analysis of feeding may both reflect and predict neurological sequelae, and perhaps points to a critical period where intervention may be most efficacious.

## Data Availability Statement

The raw data supporting the conclusions of this article will be made available by the authors, without undue reservation.

## Ethics Statement

This study was approved by the Institutional Review Boards of the University of Kentucky School of Medicine and Michigan State University College of Human Medicine. Written parental informed consent was obtained prior to studying each infant. Written informed consent to participate in this study was provided by the participants' legal guardian/next of kin.

## Author Contributions

IG: conceived, planned, designed study, analyzed and interpreted data, and wrote all drafts of the paper. BS: helped design study and analyzed and interpreted data. FV: helped design study, downloaded, analyzed and interpreted data, and reviewed manuscript. AP: managed data collection system. NS: obtained consents and enrolled patients and analyzed dataset. ER: conceived, planned and designed study, enrolled patients, analyzed and interpreted data, and assisted in writing of manuscript. All authors contributed to the article and approved the submitted version.

## Conflict of Interest

The authors declare that the research was conducted in the absence of any commercial or financial relationships that could be construed as a potential conflict of interest.

## Publisher's Note

All claims expressed in this article are solely those of the authors and do not necessarily represent those of their affiliated organizations, or those of the publisher, the editors and the reviewers. Any product that may be evaluated in this article, or claim that may be made by its manufacturer, is not guaranteed or endorsed by the publisher.

## Abbreviations

IVH: Intraventricular hemorrhage
BPD: Bronchopulmonary dysplasia
LRP: Low-risk Preterm
COV: Coefficient of Variation
PMA: Post-menstrual Age
PNA: Post-natal Age
GA: Gestational Age
BW: Birthweight
NICU: Neonatal Intensive Care Unit
NOMAS: Neonatal Oral-Motor Assessment Scale.
